# Revisiting Parakinesia Brachialis Oscitans: You Yawn, I Move! A Scoping Review

**DOI:** 10.5334/tohm.1267

**Published:** 2026-10-21

**Authors:** Rani Priyanka Vasireddy, Ricardo A. Vivanco, Aishwarya Koppanatham, Abhishek Lenka

**Affiliations:** 1 Department of Neurology, University of Texas at Tyler School of Medicine, USA; 2 Department of Neurology, University of Kentucky, USA; 3 Sri Venkateswara Institute of Medical Sciences, India; 4 Department of Neurology, Virginia Commonwealth University, USA

**Keywords:** Parakinesia Brachialis Oscitans, PBO, neuroanatomy, motor control, neuroimaging

## Abstract

**Background:**

Parakinesia brachialis oscitans (PBO) is a rarely reported phenomenon in which a paretic limb moves involuntarily during yawning. The literature consists predominantly of case reports and small series, with limited comparative evidence.

**Methods:**

We conducted a PRISMA-ScR-guided scoping review of PubMed, Embase, and Web of Science from inception to 28 August 2026, with backward citation searching. Eligible reports described one or more patients with an involuntary, stereotyped, yawn-locked movement of a paretic limb after a focal central nervous system lesion, with the lesion site reported on neuroimaging. Data included demographics, movement description, latency, lesion site, etiology, and outcome.

**Results:**

Twenty-two reports describing 31 patients met the inclusion criteria. Most patients were men (67.7%), with a mean age of 56.1 years. PBO involved the upper limb alone in 29 patients (93.5%). Acute ischemic stroke was the most common etiology (83.9%). Lesions were often seen in the internal capsule (44.4%) and the corona radiata or subcortical white matter (40.7%). Onset ranged from immediate to four months after the lesion. Complete resolution was documented in 10 patients (32.3%), PBO persisted for up to three years in five, and outcome was not reported in 15 (48.4%).

**Discussion:**

PBO is associated with focal lesions affecting distributed voluntary motor-control networks, frequently involving subcortical motor pathways. Although no mechanism has been established, the observations suggest a release of preserved brainstem and extrapyramidal circuits. Outcome was unreported in almost half the cases; prospective, standardized reporting is needed to clarify its course and prognostic significance.

**Highlights:**

- This scoping review gathered 22 reports describing 31 patients with PBO after a focal brain injury.
- PBO involved the upper limb alone in 29 patients and lesions were most commonly subcortical, involving the internal capsule.
- Onset ranged from presentation to four months and PBO persisted for up to three years. However, the outcome was not reported in half of the included cases.

## Introduction

Parakinesia brachialis oscitans (PBO) is a rarely reported neurological phenomenon in which involuntary movements emerge in a paretic limb during yawning. Although similar observations in hemiplegia were described long before the phenomenon was named, from Erasmus Darwin in 1794 and Abercrombie in the 19th century [1,2], the term *parakinesia brachialis oscitans* was formally introduced by Walusinski et al. [1]. PBO usually emerges hours to months after a precipitating brain lesion, most frequently a cerebrovascular event [1,3]. The movement is most consistently described as shoulder abduction while starting to yawn and followed by spontaneous return to rest upon completion of yawning [1,4]. While upper-limb involvement predominates, lower-extremity manifestations have been reported in a minority of cases [2,5].

Although it is a rarely reported phenomenon, PBO has meaningful clinical implications. Its stereotyped, yawn-locked pattern has to be distinguished from a focal motor seizure and from other involuntary movements of a paretic limb, a distinction that relies on clinical observation [3]. It may also be read as a return of voluntary movement: de Lima et al. described a patient who regarded his own PBO as a sign of impending functional improvement [5]. Therefore, the importance of clear communication about their nature and significance in patient counseling should be underscored.

The current literature on PBO consists predominantly of isolated case reports and small case series, with no systematic synthesis of lesion sites, etiologies, and reported course across them. This scoping review systematically identifies and synthesizes published PBO cases to characterize their clinical features, underlying etiologies, and neuroanatomical correlates, to provide a more coherent descriptive framework for this rarely reported phenomenon.

## Methods

This review was conducted and reported according to the PRISMA extension for Scoping Reviews (PRISMA-ScR) [6]; the completed checklist is provided as Supplementary File 2. No protocol was registered. PubMed, Embase, and Web of Science were searched from database inception through 28 August 2026. The search strategy used the terms “parakinesia brachialis oscitans” with a broader concept block pairing yawning with the descriptive terms for the phenomenon including associated movements, associated reactions, involuntary stretching, synkinesis, and automatic movements, in the context of limb weakness. No language, publication date, publication type, or study design limits were applied at the search stage. The search strategy returned 36 records in PubMed, 61 in Embase, and 45 in Web of Science, identifying 142 records in total. Two further reports were identified by backward citation searching of the reference lists of all included reports. The complete database-specific strategies, with field tags and Boolean operators, are provided as Supplementary Table 1.

PBO was defined operationally as an involuntary, stereotyped movement of a paretic limb described as elevation of the upper limb through shoulder abduction with elbow flexion and coupled to yawning, in a patient with hemiparesis or hemiplegia attributable to a focal central nervous system lesion. A report was eligible when all four of the following were documented: (1) the movement occurred only in association with yawning and was reproducible across yawns; (2) it involved the paretic limb or limbs; (3) it was bounded by the yawn, the limb returning to its resting paretic position as the yawn ended; and (4) it was not under volitional control when first observed. Coexisting spasticity did not exclude a case, since PBO is described in both the flaccid and the spastic stage, and a superimposed tremor did not exclude a case provided the dominant yawn-locked event was patterned limb elevation rather than a rhythmic oscillation.

We included case reports and case series whose main objective was to describe one or more patients with PBO as defined above, and which reported the lesion site on neuroimaging. We excluded reports describing a movement provoked by effort, posture, or resisted movement of the contralateral limb [7], a focal motor seizure on clinical or electrographic evidence, or an exacerbation of a pre-existing movement disorder, and patients in whom the movement occurred in a limb with no documented weakness. Narrative and systematic reviews without primary case data, animal studies, reports without a specified lesion site, and reports not published in English were also excluded.

Duplicate records were removed before screening. Titles and abstracts, and then the full texts of all potentially eligible reports, were screened independently by two authors (R.V. and A.K.). Disagreements at either stage were resolved by discussion, with a third author (R.P.V.) adjudicating where consensus was not reached. Data were extracted by two authors (R.V. and A.K.) and verified by a third (R.P.V.) using a standardized extraction form developed for this review. All included reports were compared on age, sex, side and site of lesion, etiology, and clinical presentation to identify duplicate patients; where two reports described the same patient, the more complete report was retained and the other excluded as a duplicate publication. Consistent with PRISMA-ScR guidance, no formal critical appraisal of individual sources of evidence was undertaken, as the objective was to map the range and nature of reported cases rather than to estimate an effect. The screening and selection process is presented in Figure 1.

**Figure 1:**
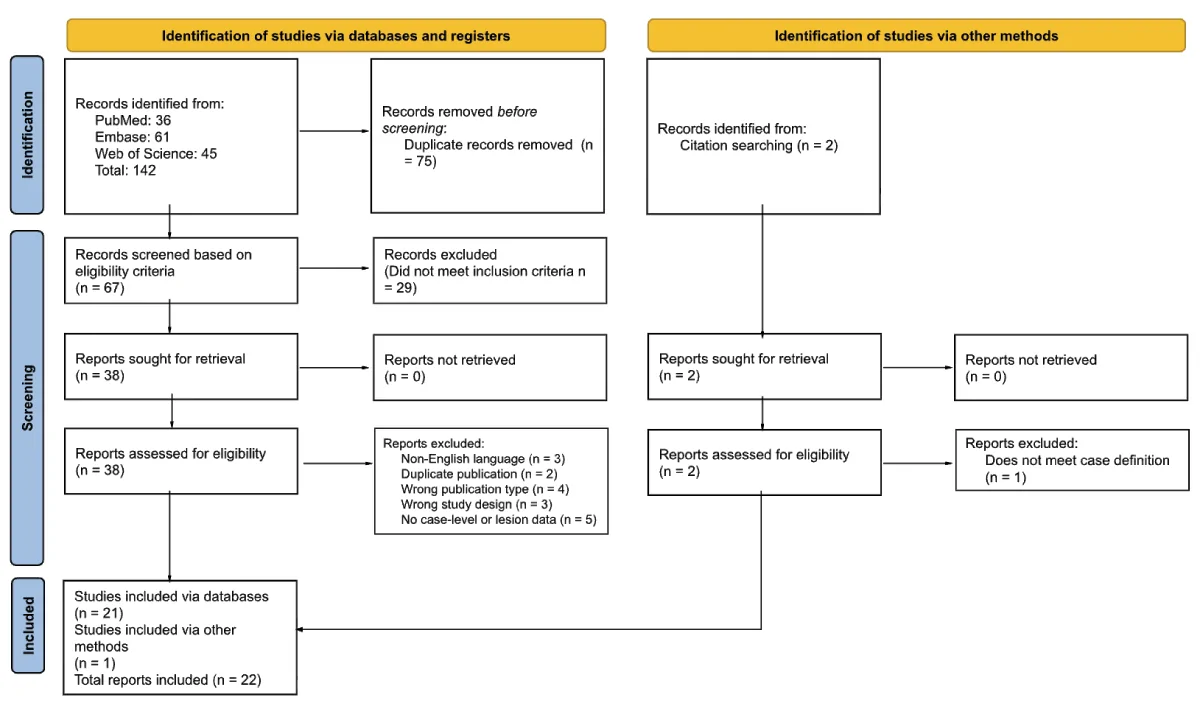
PRISMA Flowchart. Study identification, screening, and inclusion process, adapted from Page MJ, et al. BMJ 2021;372:n71. doi: 10.1136/bmj.n71.

Data collected included age and sex; initial clinical presentation, including the Medical Research Council (MRC) grade [8] in the affected limb where reported; a verbatim description of the abnormal movement; the side of the lesion and limbs involved; latency from the neurological injury to the onset of PBO; lesion location on imaging; vascular territory; final diagnosis and etiology; any treatment directed at PBO; and outcome, including time to resolution. Because the source reports use anatomical structures and vascular territories interchangeably, lesion location was coded on two independent axes: involvement at each of six anatomical levels, which are not mutually exclusive, and vascular territory, recorded only where the source authors named one and never inferred from the structures involved. Categories for latency and outcome were derived after extraction and applied uniformly to all included cases. Full definitions are provided as Supplementary Table 2.

Findings were synthesized descriptively. Continuous variables are reported as mean (standard deviation) and range, and categorical variables as n (%) with the denominator stated. No meta-analysis or statistical comparison between subgroups was undertaken, given the sample size and the descriptive aim of the review.

## Results

The systematic search identified 142 records. After 75 duplicates were removed, 67 records were screened by title and abstract and 29 were excluded. Thirty-eight reports were assessed in full text and 17 were excluded, leaving 21 studies included from databases. Citation searching identified two further records, one of which did not meet the case definition. In total, 22 reports met the inclusion criteria, 21 identified through databases and one through other methods (Figure 1). These reports describe 31 patients who met the operational definition.

### Baseline Clinical Characteristics

Across the included studies, thirty-one individual cases of PBO were described (Table 1), and aggregated data are summarized in Table 2 (extended summary available in Supplementary Table 3). Most patients were men (67.7%, n = 21), with a mean age at presentation of 56.1 years (SD 13.6; range 20–76). Weakness without any other neurological deficit was reported in three cases (9.7%), while the remaining patients presented with associated neurological features, most frequently dysarthria (32.3%) and facial weakness (32.3%), followed by aphasia (19.4%), sensory abnormalities (9.7%), headache (9.7%), limb incoordination (6.5%), reduced level of consciousness (6.5%), vertigo (3.2%), visual field deficit (3.2%) and seizures (3.2%). Grading of motor strength using the MRC scale was explicitly reported in only six cases, with upper-limb grades ranging from 0 to 4.

**Table 1:** Clinical and neuroanatomical features of reported parakinesia brachialis oscitans cases. Cases are ordered chronologically by year of publication. Full case-level data are given in Supplementary Table 3. CADASIL, cerebral autosomal dominant arteriopathy with subcortical infarcts and leukoencephalopathy; ICA, internal carotid artery; MCA, middle cerebral artery; NR, not reported; PBO, parakinesia brachialis oscitans; PCA, posterior cerebral artery.


#	AUTHOR (YEAR)	AGE/SEX	LESION/DIAGNOSIS	SYMPTOM LATERALITY	LIMB(S) AFFECTED	TIME TO ONSET	PBO OUTCOME

1	Blin et al. (1994)	62/M	Left posterior limb of internal capsule infarct	Right	Upper & Lower	A few days	Persisted at 6 weeks

2	Töpper et al. (2003)	62/M	Right PCA and MCA territory infarction	Left	Upper	NR	NR

3	Töpper et al. (2003)	43/M	Basilar artery thrombosis with right pontine infarct	Left	Upper	4 days	NR

4	Töpper et al. (2003)	51/M	Hypertensive left thalamic hemorrhage extending into the internal capsule	Right	Upper	2 weeks	NR

5	Walusinski et al. (2010)	49/F	Right internal capsule infarct in the setting of CADASIL (NOTCH3 mutation)	Left	Upper	2 days	Resolved at 2 weeks with motor recovery

6	Walusinski et al. (2010)	73/M	Left internal capsule lacunar infarct	Right	Upper	At presentation	Resolved at 1 week with partial recovery and residual ataxia

7	Walusinski et al. (2010)	71/M	Right centrum semiovale and lenticular nucleus infarct	Left	Upper	At presentation	Persisted at 3 years

8	Walusinski et al. (2010)	53/M	Right caudate and centrum semiovale infarct in the setting of factor V Leiden	Left	Upper	At presentation	Persisted at 1 year

9	Walusinski et al. (2010)	75/M	Left internal capsule and lenticular nucleus infarct	Right	Upper	At presentation	NR

10	Walusinski et al. (2010)	35/F	Left total MCA territory infarct in the setting of ICA dissection and fibromuscular dysplasia	Right	Upper	At presentation	NR

11	de Lima et al. (2012)	63/M	Right cortical and subcortical frontoparietal infarct	Left	Upper	2 months	NR

12	Jung et al. (2012)	59/M	Right precentral gyrus and frontal subcortical infarct	Left	Upper	1 day	Resolved, timing NR, by 5-year follow-up

13	Zorzetto et al. (2013)	60/M	Left MCA territory infarct	Right	Upper	During thrombolysis	Resolved at 24 hours

14	Wu et al. (2013)	52/M	Right putaminal hemorrhage extending into the internal capsule	Left	Upper	4 months	Persisted, increasing in frequency and intensity

15	Farah et al. (2015)	51/M	Left fronto-parieto-temporal infarct	Right	Upper	2 days	Resolved at 12 hours

16	Kang and Dhand (2015)	63/M	Left cortical and subcortical infarct in the MCA territory	Right	Upper	NR	Resolved at 2 weeks with partial recovery of arm strength

17	Koo and Wang (2016)	57/F	Right internal capsule infarct	Left	Upper	A few days	NR

18	Nascimento Alves et al. (2018)	59/M	Left lenticular nucleus, internal capsule, caudate and corona radiata infarct	Right	Upper	At presentation	NR

19	Aaron and Al Hashmi (2019)	53/F	Right pontine and upper medullary infarct	Left	Upper	2 days	Resolved at 3 days

20	Gadelha et al. (2019)	65/F	Right MCA territory infarct with M1 occlusion	Left	Upper	During thrombolysis	NR

21	Chowdhury et al. (2022)	59/M	Left frontal cortex and basal ganglia infarct with hemorrhagic transformation	Right	Upper	2 weeks	Persisted at 1 year with reduced amplitude

22	Lim and Neo (2022)	65/M	Right corona radiata and internal capsule infarct	Left	Upper	17.5 hours	Resolved, timing NR, with recovery of motor power

23	Salavisa et al. (2023)	35/M	Right centrum semiovale demyelinating lesion; multiple sclerosis	Left	Upper	2 weeks	Improved, then re-emerged with neurological worsening

24	Zobiri et al. (2023)	71/F	Internal capsule and lenticular nucleus infarct, right MCA territory	Left	Upper	NR	NR

25	Tsai et al. (2024)	31/M	Right deep subcortical white matter tumefactive demyelinating lesion	Left	Upper	At presentation	NR

26	Alvarez and Alvarez (2025)	76/M	Right frontal cortical-subcortical intracerebral hemorrhage	Left	Upper	NR	NR

27	Orozco-Puga et al. (2026)	20/F	Right internal capsule infarct in the setting of ICA dissection	Left	Upper	1 week	NR

28	Orozco-Puga et al. (2026)	45/F	Left pontine infarct with hemorrhagic transformation	Right	Upper	4 days	NR

29	Orozco-Puga et al. (2026)	49/F	Right frontal subcortical white matter infarct	Left	Upper	2 weeks	NR

30	AlJaafari and Shariff (2026)	57/M	Left pontine infarct with basilar atherosclerosis	Right	Upper & Lower	1 week	Resolved by 3 months

31	Alcalá-Torres (2026)	76/F	Right lenticular nucleus and internal capsule infarct	Left	Upper	1 month	Resolved at 5 months


**Table 2:** Summary of clinical and lesion characteristics in parakinesia brachialis oscitans. Values are n (%) unless otherwise stated. Percentages are calculated on the denominator stated for each block. Associated neurological features and anatomical levels are not mutually exclusive. Onset beyond one month ranged from 2 to 4 months. Among cases resolving after more than one week, time to resolution ranged from 2 weeks to 5 months. Among cases that persisted, follow-up extended to 3 years.


VARIABLE	N (%)

**Sex (n = 31)**	

Men	21 (67.7)

Women	10 (32.3)

**Age at onset, years (n = 31)**	

Mean (SD)	56.1 (13.6)

Range	20 to 76

**Laterality of symptoms (n = 31)**	

Left	19 (61.3)

Right	12 (38.7)

**Limb involvement (n = 31)**	

Upper only	29 (93.5)

Upper and lower	2 (6.5)

**Associated neurological features (n = 31)**	

Dysarthria	10 (32.3)

Facial weakness	10 (32.3)

Aphasia	6 (19.4)

Sensory abnormalities	3 (9.7)

Headache	3 (9.7)

Limb incoordination	2 (6.5)

Reduced level of consciousness	2 (6.5)

Vertigo	1 (3.2)

Visual field deficit	1 (3.2)

Seizures	1 (3.2)

**Time to PBO onset (n = 31)**	

At presentation	9 (29.0)

1 to 7 days	11 (35.5)

More than 1 week to 1 month	5 (16.1)

More than 1 month	2 (6.5)

Not specified	4 (12.9)

**Anatomical level involved (n = 27)**	

Internal capsule	12 (44.4)

Corona radiata or subcortical white matter	11 (40.7)

Basal ganglia	8 (29.6)

Cortex	6 (22.2)

Brainstem	4 (14.8)

Thalamus	1 (3.7)

**Etiology (n = 31)**	

Acute ischemic stroke	26 (83.9)

Intracerebral hemorrhage	3 (9.7)

Inflammatory or demyelinating lesion	2 (6.5)

**Outcome (n = 31)**	

Resolved within 1 week	4 (12.9)

Resolved after more than 1 week	4 (12.9)

Resolved, timing not reported	2 (6.5)

Improved but not resolved	1 (3.2)

Persisted at last follow-up	5 (16.1)

Not reported	15 (48.4)


### Clinical Description and Duration of Parakinesia brachialis oscitans

PBO manifested in the upper limb alone in 29 cases (93.5%), while combined upper and lower limb involvement was observed in the remaining two cases. Limb involvement was left-sided in 19 cases (61.3%) and right-sided in 12 (38.7%), and was contralateral to the lesion in all 31 patients. PBO was present at the initial presentation in nine cases (29%) and within 1–7 days in an additional 11 cases (35.5%). Onset between more than one week and one month was reported in five cases (16.1%), and beyond one month in two cases (6.5%). Latency was not specified in four cases (12.9%). The longest reported latency was four months. One case reported botulinum toxin use for spasticity, followed by eventual resolution of PBO. Complete resolution was documented in ten patients (32.3%), occurring within one week in four cases and after more than one week in four, and at an unspecified interval in two. Improvement without resolution was reported in one patient (3.2%), and PBO persisted at last follow-up in five (16.1%). The longest reported persistence of PBO was three years. Outcome duration was not specified in 15 cases (48.4%).

### Lesion Etiology and Neuroanatomical Localization

Anatomical involvement could be determined in 27 of the 31 cases; in the remaining four the report named only a vascular territory. Involvement was most frequent in the internal capsule (12 cases, 44.4%) and the corona radiata or subcortical white matter in 11 (40.7%), followed by the basal ganglia in eight (29.6%), the cortex in six (22.2%), the brainstem in four (14.8%) and the thalamus in one (3.7%). A vascular territory was named by the source authors in only 12 of the 26 patients with an ischemic lesion (46.2%), and was the middle cerebral artery in eleven and the posterior and middle cerebral arteries together in one. These frequencies describe lesion involvement rather than independent anatomical localization, because categories were not mutually exclusive and several cases had multifocal lesions. The predominant underlying etiology was acute ischemic stroke (83.9%). Less frequent causes were intracerebral hemorrhage (9.7%) and multiple sclerosis or other inflammatory etiologies (6.5%).

## Discussion

In this scoping review of 22 reports describing 31 patients, PBO has been reported in patients with acute motor weakness attributable to disruption of voluntary motor control pathways. Acute ischemic stroke was the most common etiology in 26 patients. Among the 27 patients for whom an anatomical structure was reported, the lesion most often involved the internal capsule or the corona radiata and adjacent subcortical white matter. Interestingly, the onset ranged from the acute phase of injury to four months after the lesion, and the clinical course varied from self-limited resolution within one week to persistent symptoms for at least three years. However, the outcome was not reported in 15 patients. The clinical impact of this phenomenon on patients’ quality of life remains unknown, but no included report assessed it formally.

### Neuroanatomical and Pathophysiological Mechanisms

PBO has been reported almost exclusively in the setting of acute or subacute focal brain injury, involving the descending motor pathways, such as in the internal capsule. In this sample, the lesions were predominantly supratentorial and subcortical with the internal capsule involved in 44.4% and the corona radiata and adjacent subcortical white matter in 40.7% of the 27 patients. This distribution is compatible with the hypothesis that interruption of corticospinal output, rather than a single-site injury, is the shared substrate, but a case-based literature cannot establish which structures are necessary or sufficient. Reports of PBO resolving as strength improves are consistent with a state-dependent phenomenon, although this cannot be concluded from the present data: PBO persisted for one to three years in 5 patients (16.1%), complete resolution was documented in only 10 (32.3%), and outcome was not reported at all in 15 (48.4%). Observed resolution proportions should not be interpreted as estimates of the natural history of PBO because follow-up was unavailable or incompletely reported in nearly half of cases and follow-up duration varied substantially.

Lim and Neo [9] have proposed at least two hypotheses to explain how yawning triggers this release phenomenon: the emotional motor system and the proprioceptive loop theories (Figure 2). Neither has been tested directly in patients with PBO. The emotional motor system theory highlights that yawning is a coordinated somatic expression of emotional disengagement, leading to co-activation of bulbar and motor neurons. These neurons allow involuntary, stretch-like limb movements via intact reticulospinal pathways when the corticospinal tract is injured [10,11]. The proprioceptive loop theory proposes that yawning induces a contraction of the respiratory muscles, which sends afferent signals through the ventral spinocerebellar tract to the spinal cord, cerebellum, and the lateral reticular nucleus in the medulla. This triggers an efferent response that travels along extrapyramidal pathways back to the cervical spinal cord (C4–C8), which may produce involuntary upper-limb movement [1]. However, this explanation does not fully account for lower limb involvement reported in some cases, a limitation raised by de Lima et al [5]. Lastly, some studies indicate that yawning is associated with monoaminergic activation in the brainstem, which may facilitate residual motor responses in patients with corticospinal tract injuries [12,13]. Lim and Neo proposed that dopaminergic agents may support recovery in patients with PBO in whom spinoreticular-cerebellar pathways are intact. However, this benefit has not been translated into post-stroke rehabilitation studies [9]. Moreover, it remains unclear why not all patients with focal brain injury and limb weakness develop PBO.

**Figure 2:**
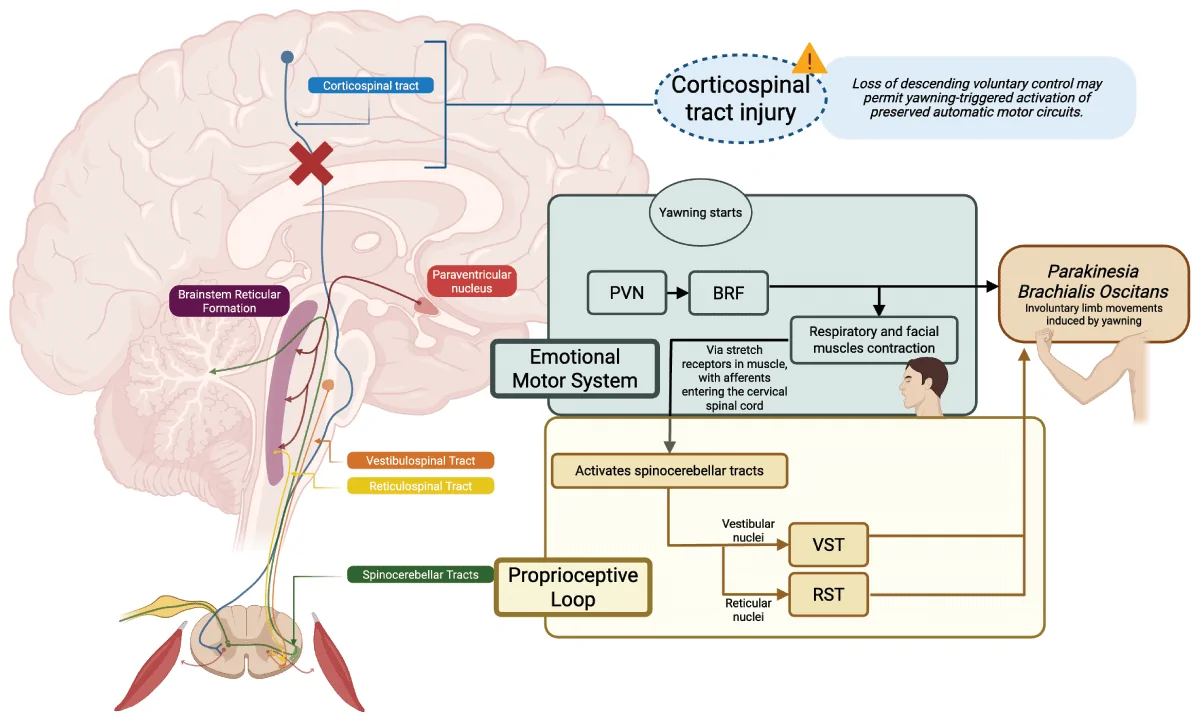
Proposed mechanistic framework for parakinesia brachialis oscitans. In a limb already weakened by a focal lesion interrupting descending voluntary control, yawning is accompanied by involuntary movement that may reflect activation of preserved automatic motor circuits. Two hypothesized pathways are illustrated. Neither has been tested directly in patients with PBO. In the emotional motor system pathway, yawning is accompanied by activation of the paraventricular nucleus (PVN) and the brainstem reticular formation (BRF), with contraction of respiratory and facial muscles, and involuntary limb movements via intact reticulospinal projections. In the proprioceptive loop pathway, muscle stretch receptors in respiratory and limb muscle and tendon send afferent signals into the cervical spinal cord and ascend in the spinocerebellar tracts, which in turn engage the vestibular and reticular nuclei, with descending motor output through the vestibulospinal tract (VST) and reticulospinal tract (RST). Both pathways are proposed routes to the involuntary yawning-triggered limb movements characteristic of PBO. BRF, brainstem reticular formation; PVN, paraventricular nucleus; RST, reticulospinal tract; VST, vestibulospinal tract.

The common denominator in both theories is an imbalance of the descending control pathways. Töpper et al. described involuntary stretching during yawning as an automatic motor pattern normally suppressed by an intact corticospinal tract, reappearing when that tract is interrupted, characteristically as spasticity and pathological reflexes return [10]. This timing is not universal: in a prospective series of 75 patients with internal capsule stroke, yawning-associated limb movements were more frequent in those with hypotonia than hypertonia (87% versus 61%) [2]. Blin et al. reached a comparable conclusion pharmacologically: apomorphine-induced yawning reproduced the same stereotyped arm movement in their patient, which they attributed to release of basal ganglia projections onto brainstem motor centers, while a simulated yawn and passive head and neck movement did not [14]. Töpper et al. noted that an intact basal ganglia projection is nonetheless not a prerequisite, since yawning with associated limb stretching has been observed after bilateral midbrain infarction [10]. A comparable release of an automatic motor pattern has been demonstrated experimentally in chronic hemiparetic stroke, where head rotation modulates flexion synergy torques in the paretic limb, reproducing the asymmetric tonic neck reflex and implicating increased reliance on ipsilateral reticulospinal pathways once corticospinal drive is lost [15]. Consistent with a proximally weighted brainstem drive, yawning-associated movements in hemiplegic limbs occur far more often at proximal than distal joints [2]. PBO is compatible with an altered interaction between impaired corticospinal control and relatively preserved brainstem and extrapyramidal pathways, although the specific circuit remains unidentified.

### Clinical Implications of Parakinesia Brachialis Oscitans

PBO is brief and occurs only with a yawn, so whether it is detected depends largely on someone being present when the patient yawns; it is likely documented most often in stroke units and inpatient rehabilitation wards. The small number of published cases therefore does not accurately estimate the prevalence of the phenomenon. Although not specifically in PBO, a prospective observational study found yawning-associated movements of the hemiplegic limbs in 78.6% of the 75 patients with internal capsule stroke, a figure close to that of an earlier survey of 40 hemiplegic patients [2,7]. Recognition of the stereotyped, yawn-locked pattern and return of the limb to rest is important for an accurate diagnosis and differentiation from other involuntary movements of a paretic limb (Supplementary Table 4) or yawning-related movement disorders (Supplementary Table 5) [16]. Importantly, the phenomenology reported under the term PBO is itself broader than the classic description: de Lima et al. reported yawning-induced upper limb movement in one patient in whom only the lower limb was paretic and in another in whom no hemiparesis was present, describing both as variants [5].

Appropriate counseling for patients and caregivers is also important to avoid leading to false expectations of functional improvement, which has not been established. Although initially noted that PBO does tend to diminish as motor recovery occurs [1], associated movements in the hemiplegic arm were subsequently judged to have no prognostic value [2]. A recent retrospective study has now examined whether PBO carries prognostic information: among patients with acute ischemic stroke matched on baseline severity, those with PBO had higher motor recovery scores at three months than those without, and PBO resolving within one month was associated with higher scores than persistent PBO [17]. Although promising, PBO cannot yet be used as a prognostic marker until prospective data are available.

### Strengths, Limitations, and Future Directions

This scoping review represents, to our knowledge, the most comprehensive synthesis of published PBO cases to date. We provided a structured overview of the characteristic presentation, lesion localization, timing, and reported course of this phenomenon. Its limitations arise from the evidence base and from the review process. The evidence base is small and unevenly reported: motor severity, imaging detail and follow-up were documented inconsistently, and lesion site was frequently given only as a vascular territory, which restricted anatomical coding to 27 of the 31 patients. Sex distribution, laterality, latency and outcome are reported as observed frequencies, without inference about sex or hemispheric predominance. Because case reports favor unusual or well-documented presentations, the published literature is unlikely to reflect either the true frequency of PBO or the range of its outcomes. Within the review process, inclusion was restricted to English-language reports, which excluded three reports and may have introduced a language bias to which a small evidence base is particularly vulnerable; no formal critical appraisal was undertaken, consistent with PRISMA-ScR guidance.

Future studies should emphasize prospective case follow-up, standardized reporting, and advanced neuroimaging approaches, such as diffusion and functional connectivity techniques, to better elucidate the neural substrates of yawning-associated motor release. While no treatments have been shown to modify PBO, rehabilitative strategies that reinforce voluntary motor control, such as mirror therapy, may warrant exploratory investigation.

## Conclusion

PBO is a rarely reported yawning-induced motor phenomenon characterized by stereotyped, involuntary limb movements associated with focal lesions affecting distributed voluntary motor-control networks, frequently involving subcortical motor pathways. The literature suggests a release-facilitated activation of brainstem and extrapyramidal pathways following loss of corticospinal control, although no mechanism has been established. Clinically, recognition of PBO is important to avoid misdiagnosis as focal seizures or misinterpretation as evidence of returning volitional movement. Improved recognition and systematic reporting will be critical to improving understanding of PBO mechanisms, prognosis, and likely implications for neurorehabilitation.

## Additional Files

The additional files for this article can be found as follows:

10.5334/tohm.1267.s1 Supplementary File 1. Supplementary Tables 1–5.

10.5334/tohm.1267.s2 Supplementary File 2. PRISMA-ScR Checklist.
