# Supplementary material for: Revisiting Parakinesia Brachialis Oscitans: You Yawn, I Move! A Scoping Review
Source: Tremor Other Hyperkinet Mov (N Y). 2026 Oct 21;16:57. doi: 10.5334/tohm.1267 (PMC13693584; doi:10.5334/tohm.1267)
Supplement: Supplementary file 1 [file tohm-16-1-1267-s1.pdf]

Supplementary Tables

Supplementary Table 1. Database search strategies and records retrieved on August 28 2026.

Source	Search strategy as executed	Records retrieved
**PubMed (MEDLINE)**	("parakinesia brachialis oscitans"[tiab]<br>OR "parakinesis brachialis oscitans"[tiab])<br>OR<br>(("yawn*"[tiab] OR yawning[mh])<br>AND<br>("abnormal movement*"[tiab] OR "involuntary movement*"[tiab]<br>OR "associated movement*"[tiab] OR "associated reaction*"[tiab]<br>OR "involuntary stretch*"[tiab] OR synkinesi*[tiab]<br>OR "automatic movement*"[tiab] OR hemipleg*[tiab]<br>OR hemipares*[tiab] OR "paretic limb"[tiab]<br>OR "paretic arm"[tiab]))	36
**Embase**	('parakinesia brachialis oscitans':ti,ab<br>OR 'parakinesis brachialis oscitans':ti,ab)<br>OR<br>(('yawn*':ti,ab OR 'yawning'/exp)<br>AND<br>('abnormal movement*':ti,ab OR 'involuntary movement*':ti,ab<br>OR 'associated movement*':ti,ab OR 'associated reaction*':ti,ab<br>OR 'involuntary stretch*':ti,ab OR 'synkinesis':ti,ab<br>OR 'synkinesia':ti,ab OR 'automatic movement*':ti,ab<br>OR hemipleg*:ti,ab OR hemipares*:ti,ab<br>OR 'paretic limb':ti,ab OR 'paretic arm':ti,ab))	61
**Web of Science**	TS=("parakinesia brachialis oscitans"<br>OR "parakinesis brachialis oscitans")<br>OR<br>TS=((yawn*)<br>AND<br>("abnormal movement*" OR "involuntary movement*"<br>OR "associated movement*" OR "associated reaction*"<br>OR "involuntary stretch*" OR synkinesi*<br>OR "automatic movement*" OR hemipleg* OR hemipares*<br>OR "paretic limb" OR "paretic arm"))	45
**Databases, total**	Sum of the three searches above, before removal of duplicates	142

Supplementary Table 2. Variable definitions and coding rules.

Domain	Variable	Definition	Values and coding rules
**Demographics**	Age	Age in years at the time of the causative lesion.	Integer as reported.
	Sex	Sex as reported by the source authors.	Male; female.
**Clinical presentation**	Motor deficit	Description of the weakness given by the source authors.	Verbatim term (hemiplegia, hemiparesis, brachiocephalic hemiplegia); not specified where no term was given.
	MRC grade	Medical Research Council grade in the affected limb, where reported.	Grade 0–5, recorded separately for upper and lower limb; not reported otherwise. Not imputed from narrative descriptions of severity.
	Associated neurological features	Deficits other than limb weakness present at the initial presentation.	Present or absent for each of dysarthria, facial weakness, aphasia, sensory abnormality, headache, limb incoordination, vertigo, reduced level of consciousness, visual field deficit and seizures. Not mutually exclusive.
**Movement**	Movement description	Description of the yawning-triggered movement.	Retained verbatim; not specified where the authors reported only that PBO occurred.
	Limbs involved	Limbs in which the movement was observed.	Upper only; upper and lower; lower only.
	Lesion side	Side of the causative lesion on neuroimaging.	Left; right; not lateralized. Recorded separately from the side of the affected limb.
	Side of affected limb	Side of the limb showing the movement.	Left; right; bilateral.
**Latency**	Time to onset of PBO	Interval from the causative insult to the first observation of PBO. Categories were derived after extraction from the range of values reported, then applied uniformly. Each interval is inclusive of its upper limit.	At presentation: at or within 24 hours of presentation, including PBO first observed during or immediately after thrombolysis.<br>1–7 days.<br>>1 week to 1 month: after 7 and up to 30 days; "one month after" is coded here.<br>>1 month.<br>Not specified: including descriptions such as "during admission" without further detail.
**Lesion location**	Anatomical level	Presence or absence of involvement at each of six levels: cortex; corona radiata and subcortical white matter; internal capsule; basal ganglia; thalamus; brainstem.	A single lesion may involve more than one level. Reports naming only a vascular territory, with no parenchymal structure specified, are coded not reported at every level and excluded from the anatomical denominator. Where the authors used a composite whole-territory descriptor, the levels conventionally encompassed by that descriptor were coded as involved.
	Vascular territory	Arterial territory of the lesion as named by the source authors.	Recorded only where the authors named a territory; never inferred from the anatomical structures involved. Not applicable where the lesion was hemorrhagic or inflammatory. Not stated where a vascular lesion was described without a named territory.
	Etiology	Underlying cause of the lesion.	Acute ischemic stroke; intracerebral hemorrhage; inflammatory or demyelinating lesion.
**Treatment and outcome**	Treatment directed at PBO	Any intervention given with the stated intention of treating the yawning-triggered movement.	Agent as reported; none where no such treatment was described. Treatment of the underlying lesion, or of a separate concurrent problem, is not counted here.
	Outcome	Course of PBO at the last reported follow-up. Categories were derived after extraction, then applied uniformly.	Resolved within 1 week: complete resolution, stated interval ≤7 days.<br>Resolved after >1 week: complete resolution, stated interval >7 days; the interval is recorded alongside.<br>Resolved, timing not specified: complete resolution described without a stated interval, including "gradually disappeared" and "faded away".<br>Improved, not resolved: reduction in frequency, amplitude or intensity without resolution.<br>Persistent at last follow-up: including cases in which the phenomenon increased over time; duration of follow-up recorded alongside.<br>Not reported.

Supplementary Table 3. Full case-level clinical and neuroanatomical data for reported parakinesia brachialis oscitans cases.

Case numbers correspond to Table 1. Movement descriptions are those given by the source authors; NR indicates that the item was not reported. The region is the country of the reporting institution. Vascular territory was recorded only where the source authors named one and was never inferred from the structures involved. For case 31 One case reported botulinum toxin use, followed by eventual resolution of PBO; the indication is not stated in the source. CTA, computed tomography angiography; DVT, deep vein thrombosis; ICA, internal carotid artery; LL, lower limb; MCA, middle cerebral artery; MRC, Medical Research Council; NIHSS, National Institutes of Health Stroke Scale; NR, not reported; PBO, parakinesia brachialis oscitans; PCA, posterior cerebral artery; PFO, patent foramen ovale; TIA, transient ischemic attack; UL, upper limb.

#	Author (Year)	Age/Sex	Initial presentation	Movement description	Laterality	Limb(s)	Time to onset	Imaging findings	Vascular territory	Final diagnosis / etiology	PBO outcome	Medical history	Region
1	Blin et al. (1994)	62/M	Right hemiplegia (UL 0/5; LL 3/5)	Shoulder abduction, anteroflexion and internal rotation with elbow flexion, extension of the first three fingers and slight leg extension	Right	Upper & Lower	A few days	Infarct in the left posterior limb of the internal capsule	NR	Left posterior limb of internal capsule infarct	Still noticeable at 6 weeks, with no recovery of the hemiplegia	NR	France
2	Töpper et al. (2003)	62/M	Left hemiparesis and hemianopia	Spontaneous movement, not further described	Left	Upper	NR	Right PCA territory infarction and extensive infarction in the right MCA territory, with swelling	Right PCA and MCA	Right PCA and MCA territory infarction	NR	NR	Germany
3	Töpper et al. (2003)	43/M	Left hemiplegia with clouding of consciousness, unequal pupils and restricted extraocular movements	Abduction and extension	Left	Upper	4 days	Thrombotic occlusion of the upper basilar artery; circumscribed right-sided lesion in the middle pons extending to the cerebellar peduncle	NR	Basilar artery thrombosis with right pontine infarct	NR	NR	Germany
4	Töpper et al. (2003)	51/M	Central facial paresis, dysarthria and complete right hemiplegia	Tonic shoulder abduction with extension of the forearm and fingers	Right	Upper	2 weeks	Hemorrhage centered in the left thalamus, extending into the posterior internal capsule	NR	Hypertensive left thalamic hemorrhage	NR	Uncontrolled arterial hypertension	Germany
5	Walusinski et al. (2010)	49/F	Left hemiplegia (UL > LL), dysarthria	Limb rise, without hand or finger movement	Left	Upper	2 days	Small infarct in the posterior limb of the right internal capsule; marked leukoaraiosis	NR	Right internal capsule infarct; CADASIL (NOTCH3 mutation)	Resolved at 2 weeks with motor recovery	Migraine with aura, smoking, family history of stroke	Switzerland
6	Walusinski et al. (2010)	73/M	Right sensorimotor hemiparesis, complete UL paralysis	Limb rise of about 30 cm	Right	Upper	At presentation	Small infarct in the anterior limb of the left internal capsule, suggesting lacunar infarction	NR	Left internal capsule lacunar infarct	Resolved at 1 week with partial recovery and residual ataxia	Hypertension, hypercholesterolemia	Switzerland
7	Walusinski et al. (2010)	71/M	Sudden vertigo followed by severe left hemiplegia	Limb rise with arm adduction and elbow flexion	Left	Upper	At presentation	Hypodensity in the right centrum semiovale and lenticular nucleus, with leukoaraiosis	Right MCA	Embolic occlusion of the lenticulostriate branches of the right MCA	Persisted at 3 years	Prior TIA, bilateral ICA stenosis, diabetes, hypercholesterolemia	France
8	Walusinski et al. (2010)	53/M	Severe left hemiplegia, then increasing drowsiness	Limb rise with arm adduction and elbow flexion	Left	Upper	At presentation	Right caudate and centrum semiovale infarct	Right MCA	Right MCA infarct with dolichoectatic carotid siphon; factor V Leiden	Persisted at 1 year	Smoking, hypercholesterolemia, factor V Leiden with prior DVT, prior TIAs	France
9	Walusinski et al. (2010)	75/M	Right brachiocephalic hemiplegia, Broca aphasia	Limb rise with arm adduction and elbow flexion	Right	Upper	At presentation	Left internal capsule and lenticular nucleus infarct	NR	Left internal capsule and lenticular nucleus infarct	NR	Alcohol use, prior TIA, bilateral carotid stenosis	France
10	Walusinski et al. (2010)	35/F	Severe headache 2 months post partum, then complete right hemiplegia, aphasia and drowsiness	Limb rise with elbow flexion, the hand moving toward the face	Right	Upper	At presentation	Total MCA territory infarct on the left; complete left ICA occlusion	Left MCA	Left MCA infarct; left carotid dissection and fibromuscular dysplasia	NR	Post partum	France

11	de Lima et al. (2012)	63/M	Left hemiparesis (UL > LL, 1/5 distal and 2/5 proximal), mild dysarthria	Abduction and elevation of the whole upper limb	Left	Upper	2 months	Right cortical and subcortical frontoparietal infarct; 30 to 40% bilateral ICA stenosis	Right MCA	Right MCA territory infarct	NR	Hypertension	Brazil
12	Jung et al. (2012)	59/M	Left hemiplegia (MRC 2/5 UL; 3/5 LL), mild dysarthria	Limb rise to chest level with arm adduction and elbow flexion, lasting about 3 seconds	Left	Upper	1 day	Right precentral gyrus and frontal subcortical infarct, without internal capsule or basal ganglia involvement	NR	Right precentral gyrus and frontal subcortical infarct	Persisted through the 17-day admission; absent at 5-year follow-up, exact date not recalled	Hypertension, head trauma 10 years earlier	Republic of Korea
13	Zorzetto et al. (2013)	60/M	Right hemiplegia and aphasia (NIHSS 15)	Limb rise with arm adduction and elbow flexion	Right	Upper	During thrombolysis	Normal CT on admission; follow-up CT showed a large ischemic stroke in the left MCA territory	Left MCA	Left MCA territory infarct	Occurred repeatedly during the initial 24 hours, then ceased	Hypertension, myocardial infarction 10 years earlier	Brazil
14	Wu et al. (2013)	52/M	Left hemiplegia (1/5 UL; 2/5 LL) after acute loss of consciousness	Progressive shoulder abduction, anteroflexion and internal rotation, then arm lifting with elbow flexion	Left	Upper	4 months	Right putaminal hemorrhage (3 x 5 cm) extending into the posterior internal capsule, with mass effect	NR	Right putaminal hemorrhage	Frequency and intensity increased steadily and the movement also occurred when sitting	Hypertension	Taiwan
15	Farah et al. (2015)	51/M	Right hemiparesis (1/5 proximal, 2/5 distal UL; 3/5 LL), central facial paralysis and motor aphasia	Lifting of the entire upper limb with tremor, returning to the resting paretic position when yawning ceased	Right	Upper	2 days	Large infarct in the left fronto-parieto-temporal area; left ICA and MCA occlusion	Left MCA	Left MCA territory infarct; atrial fibrillation with left ventricular thrombus	Ceased after 12 hours	Hypertension, dyslipidemia, type 2 diabetes, obesity, prior stroke	Brazil
16	Kang and Dhand (2015)	63/M	Right arm paralysis (MRC 0/5) with the leg spared, and aphasia	Limb rise to the chest	Right	Upper	NR	Cortical and subcortical involvement in the left MCA vascular territory	Left MCA	Left MCA territory infarct	Ceased after 2 weeks following partial recovery of arm strength (MRC 4)	NR	USA
17	Koo and Wang (2016)	57/F	Acute left hemiplegia affecting face, arm and leg	Arm abduction and flexion, not suppressible and not reproducible voluntarily	Left	Upper	A few days	Acute infarct in the posterior limb of the right internal capsule	NR	Right internal capsule lacunar infarct	NR	Multiple sclerosis, diabetes, hypertension	NR
18	Nascimento Alves et al. (2018)	59/M	Right hemiplegia (MRC 0/5 UL), pain anesthesia, dysarthria and facial palsy	Reflexive, stereotyped elbow flexion; voluntary control over the movement emerged at day 6	Right	Upper	At presentation	Infarct involving the lenticular nucleus, anterior and posterior limbs of the internal capsule, body of the caudate nucleus and corona radiata	Left MCA	Left MCA infarct treated with thrombolysis	NR	Diabetes mellitus, arterial hypertension	Portugal
19	Aaron and Al Hashmi (2019)	53/F	Left hemiparesis (1/5 UL; 0/5 LL) with mild right lower motor neuron facial weakness	Limb rise above the head	Left	Upper	2 days	Acute infarct in the right pons and upper medulla; extensive intracranial atherosclerosis	NR	Right pontine and upper medullary infarct	Observed for 3 days and gone by discharge	Poorly controlled diabetes and hypertension	Oman
20	Gadelha et al. (2019)	65/F	Acute dysarthria, left-sided weakness and numbness	Elbow and wrist flexion with subtle shoulder abduction	Left	Upper	During thrombolysis	Large stroke in the right MCA distribution; CTA showed a right M1 occlusion	Right MCA	Right MCA territory infarct	NR	Smoking	Brazil
21	Chowdhury et al. (2022)	59/M	Right hemiparesis (MRC 2/5 UL; 3/5 LL), motor aphasia and facial weakness	Progressive shoulder abduction with elbow flexion and mild supination, and occasional wrist dorsiflexion	Right	Upper	2 weeks	Chronic infarction in the left lateral frontal cortex and white matter extending to the operculum, precentral gyrus and basal ganglia, with hemorrhagic transformation	Left MCA	Left MCA territory infarct with hemorrhagic transformation	Still present at 1 year, with a decreased degree of lifting	Diabetes, hypertension, 30 pack-year smoking	India
22	Lim and Neo (2022)	65/M	Left hemiplegia, dysarthria, facial droop and hemiataxia (NIHSS 7)	Antigravity shoulder abduction, only when yawning	Left	Upper	17.5 hours	Acute infarct in the right corona radiata and internal capsule; no large vessel occlusion on CTA	NR	Right corona radiata and internal capsule infarct, treated with thrombolysis	Gradually disappeared with recovery of motor power; interval not stated	Hypertension, hyperlipidemia, smoking	Singapore

23	Salavisa et al. (2023)	35/M	Generalized tonic-clonic seizure followed by left spastic hemiparesis (MRC 3/5 shoulder abduction; 2/5 wrist)	Arm abduction with distal high-amplitude postural hand tremor, not reproducible voluntarily	Left	Upper	2 weeks	47 mm oval T2-hyperintense lesion in the right centrum semiovale with C-shaped enhancement and a central vein sign, plus a frontal juxtacortical lesion	NR	Multiple sclerosis	Gradual reduction in frequency and amplitude, then re-emergence with subacute neurological worsening	Autism spectrum disorder, mild learning difficulties	United Kingdom
24	Zobiri et al. (2023)	71/F	Total left hemiplegia with dysarthria (NIHSS 15)	NR	Left	Upper	NR	Normal initial CT; CT at 24 hours showed infarction in the right MCA territory involving the internal capsule and lenticular nucleus	Right MCA	Right MCA territory infarct treated with thrombolysis	NR	Hypertension	Algeria
25	Tsai et al. (2024)	31/M	Left ataxic hemiparesis for five days, with frequent yawning	Transient tonic elevation of the paralyzed arm	Left	Upper	At presentation	Mass-like abnormality involving the deep right subcortical white matter, right corona radiata, centrum semiovale and corpus callosum	NR	Biopsy-confirmed tumefactive demyelinating lesion	NR	None; preceded by an upper respiratory tract infection	Canada
26	Alvarez and Alvarez (2025)	76/M	Acute left-sided weakness (UL 2/5; LL 1/5) with left facial asymmetry	Repetitive involuntary limb elevation, accompanied by spontaneous penile erection and not reproducible voluntarily	Left	Upper	NR	Hyperacute right frontal intracerebral hemorrhage at the cortical-subcortical medial precentral gyrus on MRI	NR	Right frontal intracerebral hemorrhage	NR	NR	Philippines
27	Orozco-Puga et al. (2026)	20/F	Sudden frontal headache followed by left-sided weakness and inability to walk	Involuntary elevation of the paretic arm	Left	Upper	1 week	Right internal carotid artery dissection with ischemic stroke involving the right internal capsule	NR	Right internal capsule infarct; carotid artery dissection	NR	Oral contraceptive use	Mexico
28	Orozco-Puga et al. (2026)	45/F	Subacute dysarthria and right hemiparesis (NIHSS 10) after chest pain and headache	NR	Right	Upper	4 days	Left hemipontine infarction with hemorrhagic transformation	NR	Left pontine infarct; embolic stroke of undetermined source with incidental PFO	NR	Hypertension after preeclampsia, type 2 diabetes, hypothyroidism, dyslipidemia	Mexico
29	Orozco-Puga et al. (2026)	49/F	Left central facial palsy and hemiparesis with ipsilateral hyperreflexia and hemiparetic gait	NR	Left	Upper	2 weeks	Right frontal subcortical white matter ischemic lesion with diffusion restriction, without hemorrhagic transformation	NR	Right frontal subcortical infarct; cryptogenic	NR	Hypertension, hypothyroidism, dyslipidemia, paternal history of stroke	Mexico
30	AlJaafari and Shariff (2026)	57/M	Right hemiparesis, dysarthria and facial weakness (NIHSS 5), progressing to 0/5 in the arm and 3/5 in the leg	Limb rise with shoulder abduction, elbow and finger flexion, and slight hip flexion, returning to the paretic position at the end of the yawn	Right	Upper & Lower	1 week	Acute infarction in the left pons alongside an old infarct; faint basilar artery irregularity on CTA	NR	Left pontine infarct with basilar atherosclerosis	Persisted through admission, reduced at 1 month and no longer noticeable by 3 months	Hypertension, smoking	Saudi Arabia
31	Alcalá-Torres (2026)	76/F	Mild left spastic hemiparesis (MRC 4/5 globally, NIHSS 3) after a right subcortical stroke	Brief (under 5 seconds) arm hyperextension with rigidity, exclusively during yawning and not reproducible voluntarily	Left	Upper	1 month	Right posterior lenticular nucleus and internal capsule lesion on MRI	NR	Right subcortical stroke	Complete resolution after 5 months of botulinum toxin therapy	NR	Spain

Supplementary Table 4. Differential Diagnosis of Yawning-Associated Limb Movements.

Phenomenon	Trigger and timing	Motor description	Course	Discriminating feature
Parakinesia brachialis oscitans	Only during a yawn; reproducible with every yawn; begins and ends with the yawn	Stereotyped patterned elevation of the paretic limb: shoulder abduction, elbow flexion, variable finger extension	Onset from presentation to months after the lesion; may resolve as strength returns or persist for years	Mandatory coupling to yawning
Associated reaction / spastic flexor synergy	Effort- and stretch-dependent: attempted voluntary movement, antigravity load, rapid passive stretch.	Sustained tonic flexion pattern (shoulder adduction with elbow, wrist and finger flexion) reflecting loss of selective motor control	Develops over weeks to months and persists or worsens	Provoked by effort or stretch, not by yawning; coexisting spasticity does not exclude PBO
Focal motor seizure	Usually spontaneous; may be sensory-provoked; may show a Jacksonian march with evolving distribution	Clonic or tonic, may spread; facial or truncal involvement; awareness may be impaired; post-ictal deficit	Recurrent, independent of yawning	March, altered awareness and post-ictal state; video-EEG capture during a yawn is definitive
Yawning-exacerbated tremor	Present independently of yawning; yawning accentuates it	Rhythmic oscillation (rest, postural or action)	Chronic, follows the underlying disorder	Rhythmic oscillation rather than a discrete patterned arm-raise; exists outside the yawn window
Physiological pandiculation	Accompanies yawning in healthy individuals	Symmetric stretching of both limbs	Lifelong, normal	Bilateral, symmetric, and occurring without limb paresis

This comparison is narrative and was not derived from the scoping-review search. Sources: Ellis MD, Schut I, Dewald JPA. Flexion synergy overshadows flexor spasticity during reaching in chronic moderate to severe hemiparetic stroke. Clin Neurophysiol. 2017;128(7):1308–14. PMID 28558314

Supplementary Table 5. Yawning-Associated Movement Disorders.

Disorder	Effect of yawning	Clinical context	Motor phenotype	Possible mechanism
Parakinesia brachialis oscitans	Triggers movement	Acute focal brain lesions	Stereotyped elevation/abduction of the paretic limb	Release of coordinated motor patterns in a weak limb following loss of descending voluntary control
Yawning-associated tremor	Exacerbates or unmasks tremor	Parkinson's disease	Transient worsening or emergence of tremor	Modulation of pre-existing tremor circuitry by autonomic activation
Prodromal tremor during yawning	Unmasks tremor	Early or prodromal Parkinson's disease	Tremor is present only during yawning	Early vulnerability of tremor networks revealed by autonomic stress
Pathological yawning	Increased yawning frequency	Stroke, MS, epilepsy, brainstem disorders	Excessive yawning without patterned limb movement	Dysregulation of yawning networks

This comparison is narrative and was not derived from the scoping-review search. Sources: Petramfar P, Jankovic J. Yawning-related tremor in patients with Parkinson's disease. Parkinsonism Relat Disord. 2026;143:108162. Teive HAG, Munhoz RP, Camargo CHF, Walusinski O. Yawning in neurology: a review. Arq Neuropsiquiatr. 2018;76:473–480. Fearon C, et al. On the Emergence of Tremor in Prodromal Parkinson's Disease. J Parkinsons Dis. 2020;11(1):261–269.
